# Decolorization and detoxification of sulfonated toxic diazo dye C.I. Direct Red 81 by *Enterococcus faecalis* YZ 66

**DOI:** 10.1186/s40201-014-0151-1

**Published:** 2014-12-24

**Authors:** Madhuri M Sahasrabudhe, Rijuta G Saratale, Ganesh D Saratale, Girish R Pathade

**Affiliations:** Department of Microbiology, Maulana Azad College, Aurangabad, MS India; Department of Biotechnology, Shivaji University, Kolhapur, MS India; Department of Environmental Biotechnology, Shivaji University, Kolhapur, MS India; Department of Biochemistry, Shivaji University, Kolhapur, MS India; H.V. Desai College, Pune, MS India

**Keywords:** Direct Red 81, *Enterococcus faecalis*, ABTS, Lignin peroxidase, Azoreductase, ADMI, GC-MS

## Abstract

Isolated *Enterococcus faecalis* YZ 66 strain shows ability to decolorize various industrial dyes among which, it showed complete decolorization and degradation of toxic, sulfonated recalcitrant diazo dye Direct Red 81 (50 mg/L) within 1.5 h of incubation under static anoxic condition. The optimum pH and temperature for decolorization was 7.0 and 40°C, respectively. Significant induction in the activity of intracellular oxidoreductive enzymes suggested its involvement in the decolorization of Direct Red 81. The biodegradation of Direct Red 81 was monitored by UV-Visible, FT-IR spectroscopy and HPLC. The final products were characterized by GC-MS and possible pathway of the degradation of the dye was proposed. The phytotoxicity assay (with respect to plants *Sorghum vulgare* and *Phaseolus mungo*) revealed that the degradation of Direct Red 81 produced nontoxic metabolites. Finally *E. faecalis* was employed to decolorize actual industrial effluent showing decolorization (in terms of ADMI value) with moderate COD and BOD reduction. Moreover the result increases the applicability of the strain for the treatment of industrial wastewaters containing dye pollutants.

**Electronic supplementary material:**

The online version of this article (doi:10.1186/s40201-014-0151-1) contains supplementary material, which is available to authorized users.

## Background

Azo dyes are xenobiotic compounds characterized by the presence of one or more azo linkages and aromatic rings [1]. They are the largest class of dyes with the greatest variety of colour. At least 3000 different varieties of azo dyes are extensively used in textile, paper, food, cosmetics and pharmaceutical industries [2]. Among various applications of synthetic dyes about 30,000 tons of different dyestuffs are produced per year worldwide [3]. Among these synthetic dyes, azo dyes are the most widely used which account for over 60% of the total number of dyes known to be manufactured [4,5]. Some investigators reported that azo dyes and their metabolites are toxic, carcinogenic and mutagenic in nature which leads to the formation of tumors and allergies besides growth inhibition of bacteria, protozoan, algae, plants and different animals [6,7].

In India textile industry is one of the greatest generators of liquid effluent pollutants which are often contaminated with harmful or poisonous substances. An estimate shows that textiles account for 14% (about 2200 dyeing industries) of India’s industrial production and around 27% of its export earnings [6]. However in India particularly for small scale textile industries, where working conditions and economic status do not allow them to treat their wastewater before disposal and they have no choice other than dumping all effluent into the main stream of water resources. Thus dyes released from the textile processing and dye stuff manufacturing industries results in increase in organic load of natural reservoirs. Pollution caused by dye effluents is mainly due to durability of dyes in wastewater, colour fastness, stability and resistance of dyes to degradation [8]. During industrial processing up to 40% of the used dyestuff are released into the process water producing highly coloured wastewater that affect aesthetics, water transparency and gas solubility in water bodies [9,10].

Several physical and chemical methods have been suggested for the treatment of dye contaminated wastewater but not widely used because of high cost, secondary pollution that can be generated by excessive use of chemicals [11]. In contrast, microbial degradation of dyes does not have similar problems so it is necessary to establish biological wastewater treatment of azo dyes [12]. Currently microbial biodegradation became a promising approach of dye treatment because of its cheaper, effective and more ecofriendly in nature [2]. Varieties of microorganisms including bacteria, fungi, yeasts, actinomycetes, algae and plants are capable of removing dyes from dye effluent, and become an inexpensive and promising tool for the removal of various dyes from textile dye effluents [6]. Among which bacterial cells represent an inexpensive and promising tool for the removal of various azo dyes from textile dye effluents. Recently a substantial amount of research has been carried out using single bacterial cultures like; *Lysinibacillus* sp. RGS; *Pseudomonas luteola*, *Bacillus fusiformis* KMK5, *Micrococcus glutamicus* NCIM-2168, and *Aeromonas hydrophila* shows very promising results for the azo dye decolorization [4,5,11,13].

*Enterococcus faecalis* is a nonmotile, facultatively anaerobic coccus and can survive very harsh environments. *Enterococcus* sp. was found to be catabolically versatile with the ability to utilize a wide range of unusual substrates such as; chlorpyrifos, pentaerythritol tetranitrate, 2,4,6-trinitrotoluene and phosphonate [14]. Biological neutralization of an alkaline effluent by an alkaliphile, *Enterococcus* faecium strain R-5 was reported earlier [15]. Recently Pingitore et al. (2012), reported the importance of *Enterococcus* sp. in dairy industries increases the biotechnological value of this strain [16]. A bacterial consortium, NAR-2 consisting of *Citrobacter freundii* A1, *Enterococcus casseliflavus* C1 and *Enterobacter* cloacae L17 was investigated for biodegradation of Amaranth azo dye (100 mg/l) in 30 min under sequential microaerophilic–aerobic condition [17]. Recently expression and characterization of an aerobic FMN-dependent azoreductase from *Enterococcus faecalis* was also reported [18].

The mechanism of microbial degradation of azo dyes involves the reductive cleavage of azo bonds (−N = N–) with the help of azoreductase under anaerobic conditions resulted into the formation of colorless solutions [11]. For the reduction of azo dyes, reduction to the anion radical occurs by a fast one-electron transfer reaction, followed by a second, slower electron transfer event to produce the stable dianion [19]. Thus the functional group of azo dye with higher electronic density might be unfavorable to this second electron transfer to form the dianion and leads to low or no decolorization [12]. Due to this reason sulfonated reactive group of azo dyes are normally considered to be more recalcitrant than carboxylated azo dyes. Some investigators reported that the rate limiting step during bacterial decolorization of sulfonated azo dyes is the permeation through the bacterial cell membrane [4,20].

In this study, we have used isolated *Enterococcus faecalis* YZ 66 to decolorize Direct Red 81 (DR 81). The isolated strain could decolorize DR 81 completely up to 500 mg/L. We have optimized various physicochemical parameters and studied enzymatic status during decolorization. Supplementation of carbon and nitrogen source on decolorization performance, identification of metabolites formed after decolorization using analytical techniques and toxicity study of metabolites formed after decolorization were systematically investigated.

## Material and methods

### Microorganism and culture conditions

*Enterococcus faecalis* YZ 66 was isolated from dye industry effluent contaminated soils [21] this strain was acclimatized with dye waste obtained from Spectrum Dyes and Chemicals Industry, Surat, India, containing DR81 in higher proportion by a method of Peppler (1979) [22]. The method included two steps: treatability test was carried out by enrichment culture technique and toxicity test in which toxicity was assessed in a series of parallel flasks with geometrically increasing concentration of DR81. Microbial growth was measured daily by turbidity. The concentration at which the toxic components inhibited growth was noted and was used as a warning of an upper concentration limit. Pure culture was maintained on the nutrient agar slants. Composition of nutrient broth and agar used for decolorization is (g/L) peptic digest of animal tissue 5, NaCl 5, beef extract 1.5, yeast extract 1.5 and pH 7.4 ± 0.2.

### Dyestuff and chemicals

The dye Direct Red 81 (DR 81) and actual dye effluent were obtained from Spectrum Dyes and Chemicals Industry, Surat, India. 2′-2′Azinobis-(3 ethylbenthiazoline-6 sulphonate) (ABTS) was purchased from Sigma Aldrich, USA. Nutrient broth dehydrated was purchased from Hi-Media, Mumbai, India. Tartaric acid, n-propanol was purchased from Qualigenes, India. All chemicals used were of the highest purity and of analytical grade.

### Decolorization studies

*Enterococcus faecalis* YZ 66 was grown for 24 h at 37°C on nutrient agar [21]. 10% inoculum (O.D_600_ 1.0) was used throughout the study [8,23]. The isolated strain was inoculated in nutrient broth to study the decolorizing ability of the culture. The dye was filter sterilized by using 0.2 μm filter (Sartorius Biolab, Germany) and added after sterilization of medium throughout the study. The dye (50 mg/L) was added immediately and incubated under static condition at 37°C. Aliquot (3 mL) of culture media was withdrawn at different time intervals and centrifuged at 6000×g for 20 min. Decolorization was monitored by measuring the absorbance of the culture at λ_max_ of the dye i.e. 511 nm and change in pH was also recorded. Sterile nutrient broth of different pH 3, 4, 5, 6, 7 and 8 was inoculated with 10% inoculum and incubated at 37°C under static condition. The dye concentration was 50 mg/L. For temperature studies sterile nutrient broth of pH 7.0 was inoculated with 10% inoculum and filter sterilized dye at 50 mg/L was added aseptically. The broth was incubated at 25°C, 30°C, 37°C, 40°C, 45°C and 50°C. All decolorization experiments were performed in triplicates. Abiotic control (without microorganism) was always included in each study.

In order to examine the effect of initial dye concentration on decolorization 50–700 mg/L of DR81 was added to the sterile nutrient broth inoculated with 10% inoculum of *Enterococcus faecalis* YZ 66 (O.D_600_ 1.0) and incubated at 37°C under static condition. The % decolorization was measured. All decolorization experiments were performed in triplicates. In each study abiotic control (without culture) was always included. The % decolorization and average decolorization rate was measured [5] as follows:

$$ \%\ \mathrm{Decolorization} = \frac{\mathrm{Initial}\ \mathrm{absorbance}-\mathrm{Observed}\ \mathrm{absorbance}}{\mathrm{Initial}\ \mathrm{absorbance}}\times 100\% $$

### Effect of supplementation of carbon and nitrogen sources on decolorization

To study the effect of carbon and nitrogen sources on decolorization of DR81, semi synthetic medium [8] having following composition was used (g/L); (NH_4_) _2_SO_4_;0.28, NH_4_Cl; 0.23, KH_2_PO_4_; 0.067, MgSO_4_.7H_2_O; 0.04, CaCl_2_.2H_2_O; 0.022, FeCl_3_.6H_2_O; 0.005, yeast extract; 0.2, NaCl; 0.15, NaHCO_3_; 1.0 and 1 ml/L of trace element solution containing (g/L) ZnSO_4._7H_2_O; 0.01, MnCl_2_.4H_2_O; 0.1, CuSO_4_.5H_2_O; 0.392, COCl_2_.6H_2_O; 0.248, NaB_4_O_7_.7H_2_O; 0.177 and NiCl_2_.6H_2_O; 0.02. It was further incorporated with different carbon and nitrogen sources (1% each) such as glucose, sucrose, lactose and starch, yeast extract, peptone, malt extract, meat extract and urea respectively. Filter sterilized dye 50 mg/L of the DR81 was added after inoculation of *Enterococcus faecalis* YZ 66 in sterilized media.

### Repeated dye decolorization in fed batch process

Decolourization medium containing 50 mg/L DR 81 was inoculated with 24 h grown cells of *Enterococcus faecalis* YZ 66. The resulting solution was then statically incubated at 37°C for the decolorization. After complete colour removal, the cells were collected, rinsed twice with sterile deionized water and transferred into a fresh decolorization medium for the second decolorization batch experiment. The same procedures were repeated seven times. All steps were done under aseptic conditions. For comparison, the repeated batch experiments were also conducted using free cells under identical experimental procedures.

### Preparation of the cell free extract

*Enterococcus faecalis* YZ 66 was grown in nutrient broth at 37°C for 24 h and centrifuged at 10,000 rpm for 20 minutes. The cell pellet was suspended in the potassium phosphate buffer (50 mM, pH 7.4) keeping sonifier output at 50 amp and giving 7 strokes each of 30 seconds with a 2 min interval at 4°C. The homogenate was centrifuged and supernatant was used as a source of enzymes. A similar procedure was followed to the cells of *Enterococcus faecalis* YZ 66 obtained after complete decolorization.

### Oxidative and reductive enzyme assays

The activities of laccase and lignin peroxidase were assayed spectrophotometrically in the cell free extract. Laccase activity was determined in a 2 mL mixture containing ABTS (10%) in 0.1 M acetate buffer pH 4.9 and measured as an increase in optical density at 420 nm [21]. Lignin peroxidase (LiP) activity was determined by monitoring the propanaldehyde formed at 300 nm in a reaction mixture of 2.5 mL containing 100 mM n-propanol, 250 mM tartaric acid and 10 mM H_2_O_2_ [13]. All enzyme assays were carried out at 37°C with reference blanks that contained all components except the enzyme to be assayed. All enzyme assays were conducted in triplicates and the average rates were calculated to represent the enzyme activity. One unit of enzyme activity was defined as a change in absorbance U/mL/min of the enzyme. NADH-DCIP reductase and azoreductase activity was carried out as per the method reported by Saratale (2013) [13].

### Decolorization of dye industry effluent

For the dye wastewater study, anaerobically digested effluent was used for further aerobic treatment by using the selected isolate in pure culture and the effluent was checked for COD and BOD [24]. For color removal efficiencies of dye wastewater the true color level independent of hue was measured using the American Dye Manufacturers’ Institute (ADMI 3WL) tristimulus filter method. This method is applicable to colored waters and wastewaters having color characteristic. The decolorization of actual dye wastewater by *Enterococcus faecalis* was determined by measuring ADMI from the aqueous solutions. ADMI removal percent (%) is the ratio between the removal ADMI value at any contact time and the ADMI value at initial concentration was calculated [5]. To understand the degree of biodegradation (mineralization) of dye wastewater reduction in chemical oxygen demand (COD) and biological oxygen demand (BOD) of the culture before and after incubation with *Enterococcus faecalis* YZ 66 was measured [24]. The nutrient medium was used as blank and similar condition was used for test.

### Analytical methods

The metabolites produced during the biodegradation of DR81 at 1.5 h i.e. after decolorization of the medium were extracted twice with equal volume of dichloromethane (DCM). The DCM extracts were pooled and evaporated at 40°C in a rotary evaporator and then transferred to a test tube [25]. The extracted residue was dissolved in a small volume of HPLC grade methanol and used for analysis. During UV visible spectral analysis, changes in absorption spectrum in the decolorized medium (400-800 nm) was recorded in comparison with the spectra of the undegraded dye [5]. HPLC analysis was performed in an isocratic system (Shimadzu SCL 10 AVP) equipped with dual absorbance detector using C-18 column with HPLC grade methanol as mobile phase at the flow rate of 1.0 mL/min for 10 min at 511 nm. The mobile phase used for TLC was composed of methanol: ethyl acetate: n-propanol: water: acetic acid (1:2:3:1:0.2 v/v) and the separation was done on precoated silica gel plates ‘Merck’. TLC plate was developed using iodine chamber [8]. Metabolites formed after decolorization of DR81 were characterized by using Fourier Transform Infrared Spectroscopy (Perkin Elmer 1000) (FTIR) Analysis was done in the mid IR region of 400-4000/Cm with 16 scan speed, the pellets prepared using spectrophotometric pure KBr (5:95) were fixed in sample holder and analysis was carried out. Extracted metabolites were subjected to FTIR. The metabolites formed after decolorization was identified by using Gas Chromatography-Mass Spectroscopy (Shimadzu GC-MS QP2010). The ionization voltage was 70 eV. Gas chromatography was conducted in temperature programming mode with a Resteck column (0.25 mmX 30 mm). The initial column temperature was 40°C for 4 min, which was increased linearly at 10°C /min up to 270°C and held at 4 min. The temperature of injection port was 275°C and GC-MS interface was maintained at 300°C. The helium was carrier gas; flow rate 1 mL/min and 30 min run time.

### Toxicity studies

Phytotoxicity tests were carried out in order to assess the toxicity of DR81 and metabolites formed after decolorization. Phytotoxicity tests were carried out at a final concentration of 400 ppm on two kinds of seeds. One from grains i.e. *Sorghum vulgare* (monocot) and second from pulses i.e. *Phaseolus mungo* (dicot), commonly cultivated in India. Phytotoxicity was conducted at room temperature (10 seeds of each) by watering separately 5 ml sample of control DR81 and its degradation products per day. Control set was irrigated using distilled water at the same time. Germination % as well as the length of plumule and radical was recorded after 7 days of incubation [8].

### Statistical analysis

Data was analyzed by one way analysis of variance (ANOVA) with Tukey- Kramer multiple comparison test. Readings were considered significant when P was ≤0.05.

## Results and discussion

### Decolorization experiment

The isolated *Enterococcus faecalis* YZ 66 was able to decolorize DR 81 within 1.5 h at a dye concentration of 50 mg/L. UV visible scan of the culture supernatant withdrawn at different time intervals indicated the decolorization and decrease in dye concentration from batch culture. Peak obtained at 511 nm disappeared after complete decolorization. The absorbance peak in the visible region disappeared indicating complete decolorization. In the UV spectra, the peak at 511 nm was replaced by new peak at 240 nm (Additional file 1: Figure S1). The absorbance peaks in the visible region disappeared indicating complete decolorization [26]. Decolorization with respect to time showed complete decolorization of the dye in 1.5 hours. There was proportionate increase in wet weight indicating growth of *E. faecalis* in the presence of dye (Additional file 1: Figure S2). There was no abiotic loss of DR 81 within 24 h incubation indicating that the decolorization of DR 81 was due to biological mechanism rather than adsorption. To confirm whether this decolorization is due to the variation in pH, change in pH was recorded which is in the range of 7.0 ± 0.2.

### Effect of physicochemical conditions on the decolorization performance

The effect of various physiochemical conditions such as pH, temperature, dye concentration, carbon and nitrogen sources on decolorization of DR 81 by *E. faecalis* was studied in detail. All parameters were studied at 37°C under static condition. 10% inoculum A_600_ 1.0 was used at a dye concentration 50 mg/L.

### Effect of pH

It was observed that pH of the media, affects the colour of the solution and the solubility of the dye and the enzymatic activity related to decolorization is also dependent on the pH. Generally bacterial cultures exhibit maximum decolorization at pH values near 7 or slightly alkaline pH values and the rate of colour removal tends to decrease rapidly at strongly acidic or slightly alkaline pH values [12,27]. *E. faecalis* showed complete decolorization of DR 81 at pH 7.0 within 1.5 h. It showed decolorization in the pH range of 5–8 while at pH 3 and 4 (about 40%) and at pH 9 and 10 about (about 30%) decolorization was observed after 24 hour of incubation (Additional file 1: Figure S3). Similar results were observed in *Micrococcus* sp. in the decolorization of 300 mg/L of Orange MR [28].

### Effect of temperature

Pearce et al., (2003) [12] reported that the rate of colour removal increases with increasing temperature within a defined range that depends upon the system. The temperature required to produce the maximum rate of colour removal tends to correspond with the optimum cell culture growth temperature which is in the range of 35-45°C. Temperature affects microbial growth, enzymes production and consequently, the percentage of decolouration. It was reported by Mathew and Madamwar, 2004 [23] that various microorganisms showed their survival at various temperatures ranging from 25-50°C. The decline in colour removal activity at higher temperature can be attributed to the loss of cell viability or to the denaturation of azoreductase enzyme. However, it has been shown that with certain whole bacterial cell preparation, azo reductase enzyme is relatively thermostable and can remain active up to temperature of 60°C over short period of time [29]. *E. faecalis* YZ 66 decolorized the dye under study in the range of 96-99% within a temperature of 30-40°C. At 30°C, 99.35% decolorization was observed while at 40°C, 98.54% decolorization was seen, thus showing negligible difference in percent decolorization at both the temperatures (Additional file 1: Figure S3). At 45°C and 50°C, 17.85 and 14.81% decolorization was observed, respectively (Additional file 1: Figure S3). Similar results was observed in *Pseudomonas aeruginosa* degrades 97% of Remazol Red (50 mg/L) at 40°C, 72% at 10°C and 82% at 30°C, respectively [30].

### Effect of initial dye concentration

**Figure 1:**
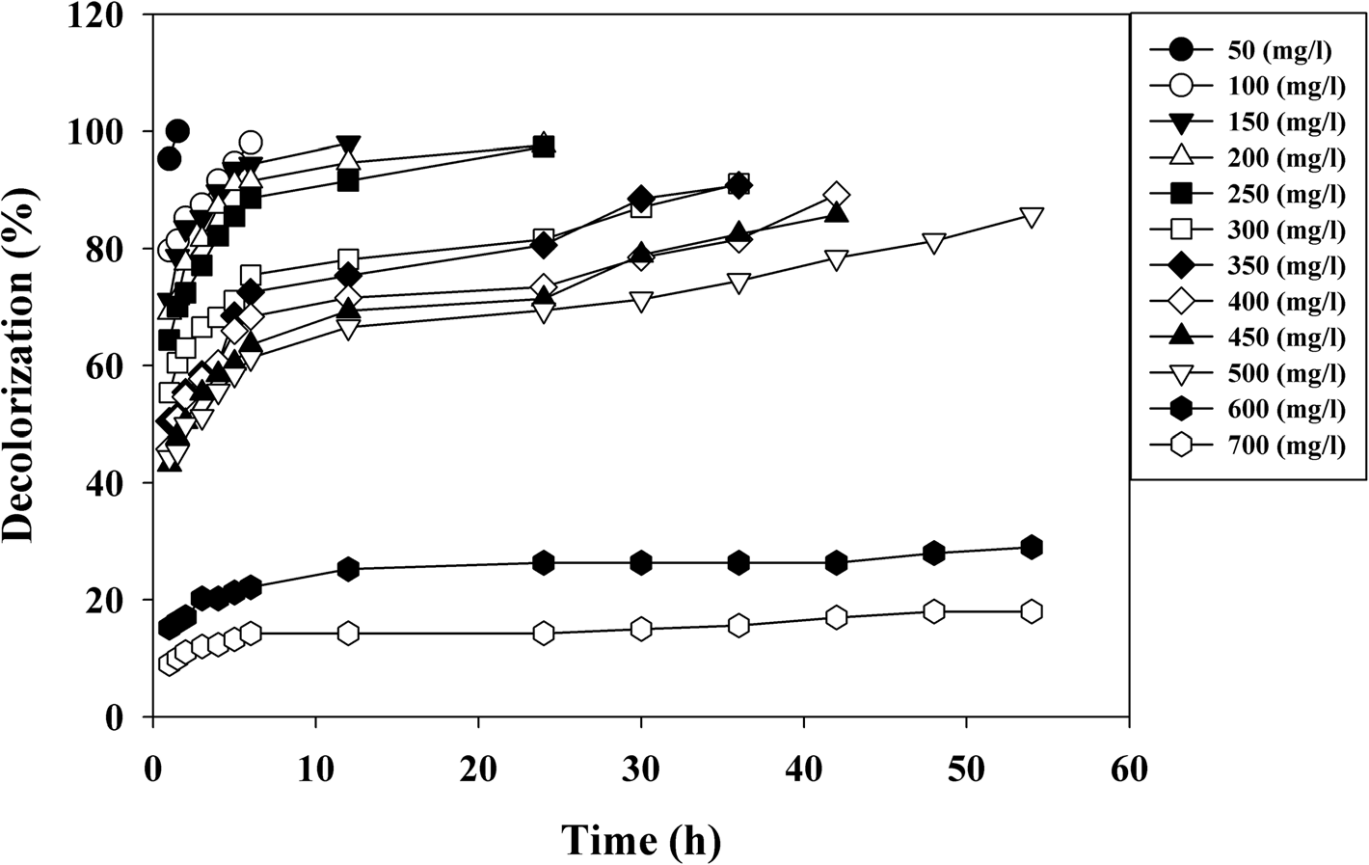
**Effect of initial dye concentration of C.I. Direct Red 81on decolorization performance by using** ***Enterococcus faecalis*** **YZ 66.**

Decolorization of different initial concentrations of the dye from 50–700 mg/L was studied under static anoxic condition. Rate of decolorization of dye increased with increase in concentration of the dye up to 300 mg/L but the time required for decolorization was more. The *E. faecalis* showed faster decolorizing ability up to 300 mg/L after which the rate of decolorization falls decreasing (Figure 1). Fifty four hours are required to decolourize 85.74% of the dye at 500 mg/L concentration. The activity was lower at dye concentration 600 mg/L and above which decolorization was strongly inhibited at dye concentration at 700 mg/L (Figure 1). It has been proposed that dye concentration can influence the efficiency of microbial decolorization through combination of factors imposed by dye at high dye concentration [31]. Similar results were observed in *Lysinibacillus* sp. (for Metanil Yellow) [32], *Sphingomonas paucimobilis* (for Methyl Red) [33], and in *Lysinibacillus* sp. RGS (for Remazol Red) [13].

### Effect of supplementation of carbon and nitrogen sources on the decolorization performance

Dyes are deficient in carbon and thus biodegradation without supplying extra carbon or nitrogen source is very difficult [34]. Carbon and nitrogen sources have an important influence on the extent of decolouration using microorganisms. In order to enhance the decolorization performance of the DR-81, an extra carbon and nitrogen source was supplied in semi synthetic medium. There was no decolorization observed in semi synthetic medium. In the presence of lactose 98.12% decolourization was observed followed by 96.16, 95.25, 95.61 and 93.76% in the presence of meat extract, peptone, glucose and starch, respectively while less decolourization with other supplements of carbon and nitrogen source within 24 h of incubation (Additional file 1: Figure S4). In addition, supplying urea as a nitrogen source did not enhance decolorizing ability. Different microbial metabolic characteristics lead to differences in the uptake of sources, thus affecting azo dye decolorization. Addition of carbon source found to be less effective to promote the decolorization performance probably due to the preference of the cells in assimilating the added carbon sources over using the dye compound as the carbon source [6,35]. Nitrogen sources are found important for microbial decolouration since it was observed that this source is essential for the regeneration of NADH [6,35].

### Decolorization with repeated addition of dye aliquots

An ability of *E. faecalis* YZ 66 to decolorize repeated addition of DR 81 dye aliquot (50 mg/L) was studied under static condition. The isolate have an ability to decolorize 100% dye up to seventh aliquot and after that subsequent cycle showed no decolorization (Additional file 1: Figure S5). The eventual cessation of decolorization was likely due to nutrient depletion [5]. Thus *E. faecalis* YZ 66 showed the ability to decolorize repeated addition of the dye aliquots is noteworthy for its commercial application.

### Enzymes involved in dye decolorization

**Figure 2:**
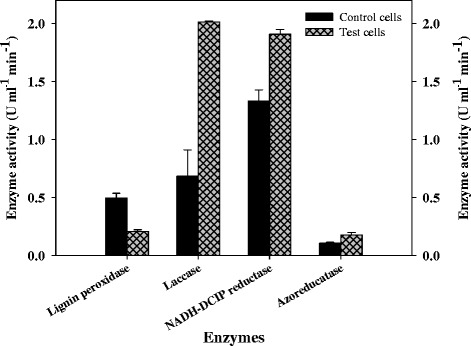
**Oxidative (lignin peroxidise and laccase) reductive (azoreductase and NADH-DCIP reductase) enzyme activity profile in control cells of** ***Enterococcus faecalis*** **YZ 66 at (0 h) and the induced cells obtained after complete decolorization of C.I. Direct Red 81 (1.5 h).**

The use of microbial techniques to deal with pollution is a key research area in the environmental sciences. In these processes microbes acclimatize themselves to the toxic wastes and resistant strains develop naturally, which then transform various toxic chemicals into less harmful forms. The mechanism behind the biodegradation of recalcitrant compounds (azo dyes) in the microbial system is based on the action of the biotransformation enzymes [36]. Besides uptake, the presence and activity of a network of detoxification enzymes is crucial for the metabolism and eventually the degradation of chemicals. To understand the decolorization mechanism, enzyme activities of laccase, lignin peroxidase, NADH-DCIP reductase, and azo reductase were monitored over time. The enhanced activities of enzymes were noted in induced cells (after decolorization) (Figure 2). The enzymatic profile presumably indicates communal action of oxidoreductive enzymes for the degradation of DR 81 into simple metabolites by *E. faecalis* (Figure 2). No enzyme activities were observed in cell free supernatant. The role of oxidoreductive enzymes in the decolorization of azo dyes have been characterized in various bacteria are well documented in recent reviews [6,37].

### Decolorization studies of dye wastewater by *E. faecalis*

**Figure 3:**
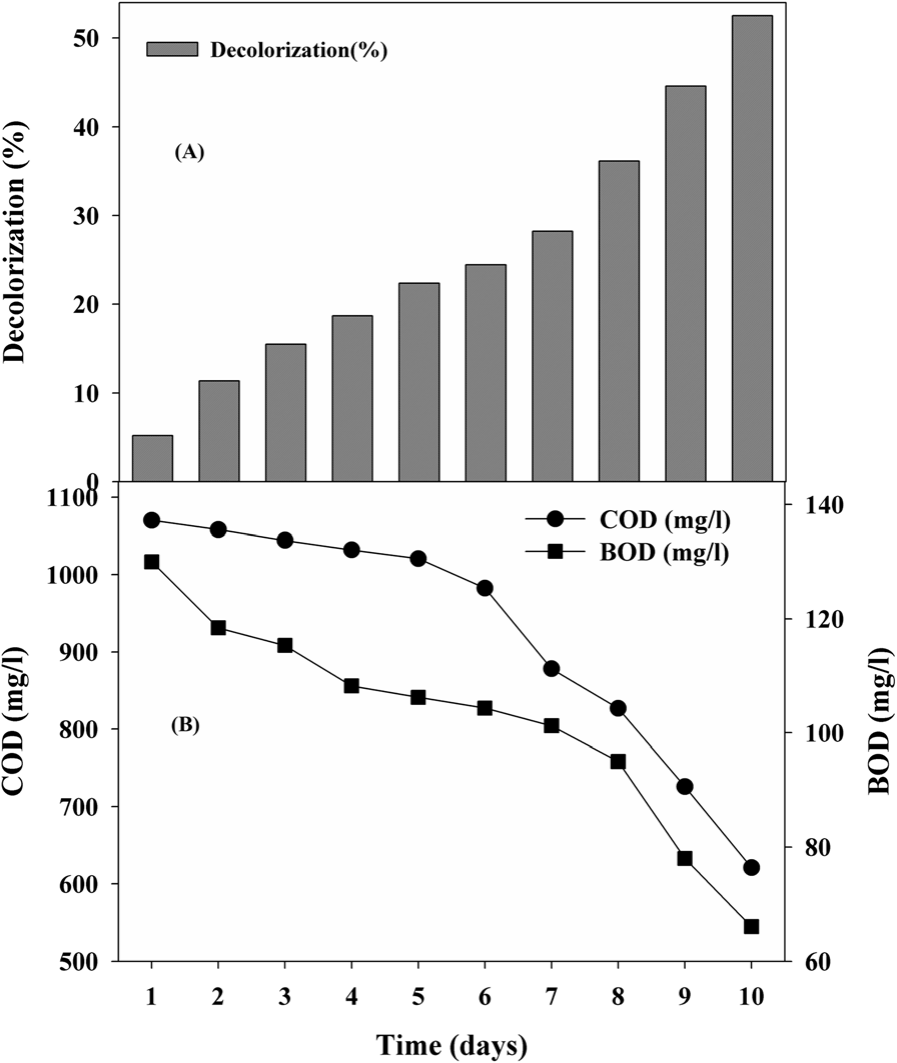
**Color removal of (A) dye industrial effluent in terms of ADMI removal ratio values (about 52%); (B) COD and BOD reduction after 10 days of incubation time by** ***Enterococcus faecalis*** **YZ 66.**

Most of the microbial decolorization studies in several laboratories showed the ability of bacteria, fungi, and algae in removing the color of textile dyes, but they do not find much application in treatment system for industrial effluent because of heterogeneity of the components in effluent depending upon production schedule. However, it is very important to test decolorization in real textile effluents, which are complex systems having strong colors, large amounts of suspended solids, broadly fluctuating pHs, high temperatures, high COD and high salt concentrations that can be inhibitory to microorganisms [13]. Considering this perspective we have checked the efficiency of *E. faecalis* to decolorize actual textile wastewater. The true color of textile wastewater measured by using ADMI 3WL suggesting that *E. faecalis* could achieve higher color removal value (52%) with moderate reduction in COD (about 42%) and BOD (about 48%) after 10 days of incubation (Figure 3). Decolorization performance of dye wastewater by *E. faecalis* is comparable with *Citrobacter* sp. strain KCTC 18061P strain removed 70% of effluent color within 5 days with 35% COD reduction [38]. Untreated dye effluents cause serious environmental and health hazards whereas in aqueous ecosystems is aesthetically unpleasant and leads to a reduction in sunlight penetration, dissolved oxygen concentration and had acute toxic effects on aquatic flora and fauna. This study is of particular relevance since the Panchganga river and Ichalkaranji area near Kolhapur, India are heavily industrialized, with significant wastewater discharge from textile and dye manufacturing industries which causes the harmful impacts to the environment. Our strain *E. faecalis* showed better colour removal of actual dye wastewater with significant reduction in COD and could be a potential strain for the treatment of textile dyestuffs and textile and dye industry effluent via appropriate bioreactor operations and will be useful to small textile industries in an ecoefficient and economically feasible that could effectively decolorize and detoxify dye containing wastewater.

### Analysis of metabolites resulting from decolorization

**Figure 4:**
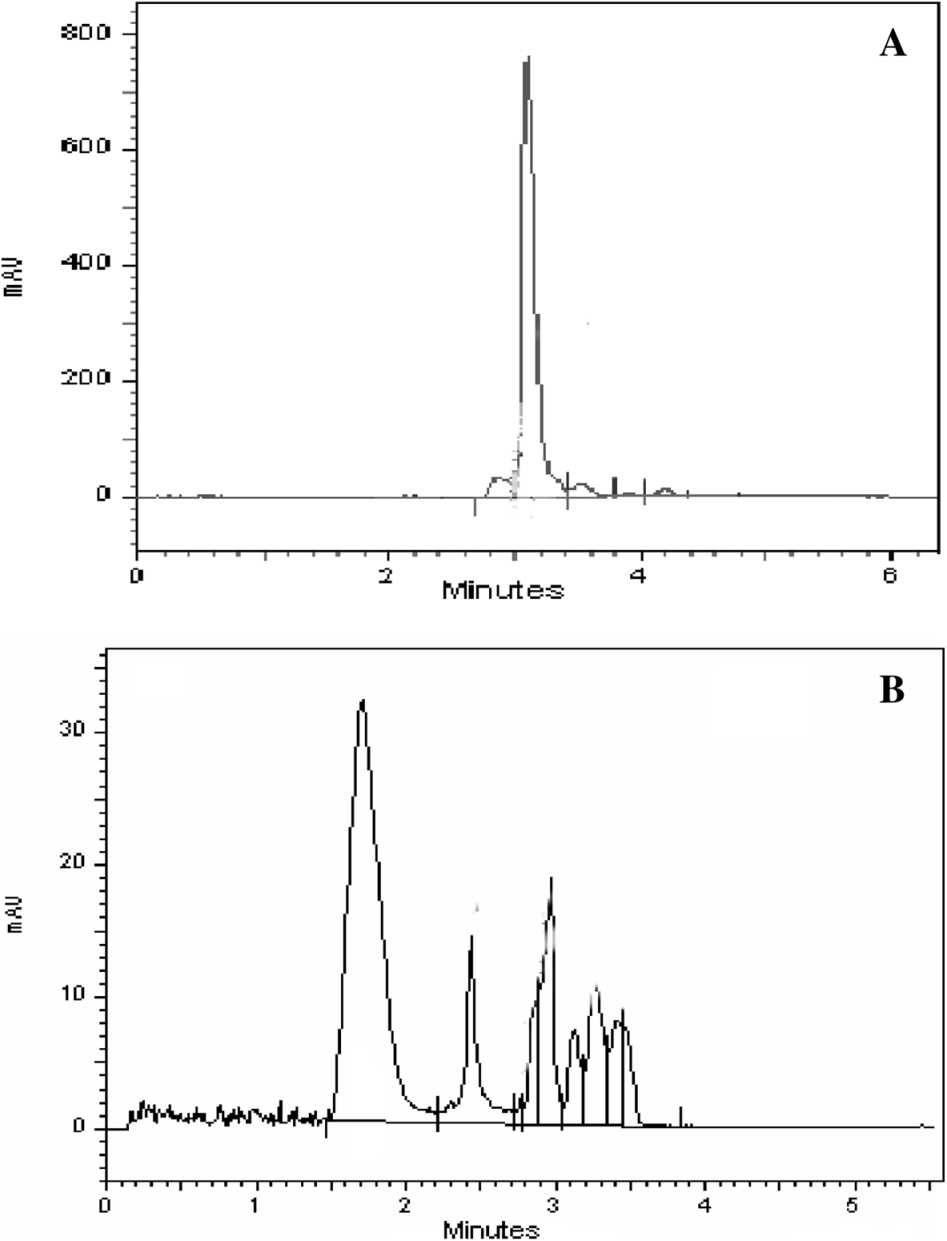
**HPLC analysis of products (extracted with ethyl acetate) formed by degradation of C.I. Direct Red 81: (A) at 0 h (control with peak at 1.71. min), (B) metabolites formed by** ***Enterococcus faecalis*** **YZ 66 after complete decolorization (1.5 h with peak at 3.008, 3.861 & 4.021 min) indicating degradation of DR 81 into different metabolites.**

To understand and confirm the possible mechanism of dye decolorization, analysis of products of biodegradation of DR 81 were studied by TLC, HPLC, FTIR and GC-MS. TLC analysis showed the appearance of one spot in the sample containing the extracted metabolites of completely decolorized medium with R_f_ value 0.71 where as R_f_ value of DR 81 was noted as 0.97 confirming the biodegradation of DR 81 by *E.faecalis* YZ 66. HPLC elution profile of DR81 showed a distinct single peak at retention time of 1.71. min. Three peaks at retention time of 3.008, 3.861 and 4.021 min were showed that the degradation of DR 81 into different products by *E. faecalis* YZ 66. Disappearance of a distinct peak of DR81 confirmed the degradation of the dye. HPLC analysis of metabolites formed after biodegradation of DR 81 showed the peaks with different retention times than the original dye which indicates the biodegradation of DR 81 into different metabolites (Figure 4A and B).

**Figure 5:**
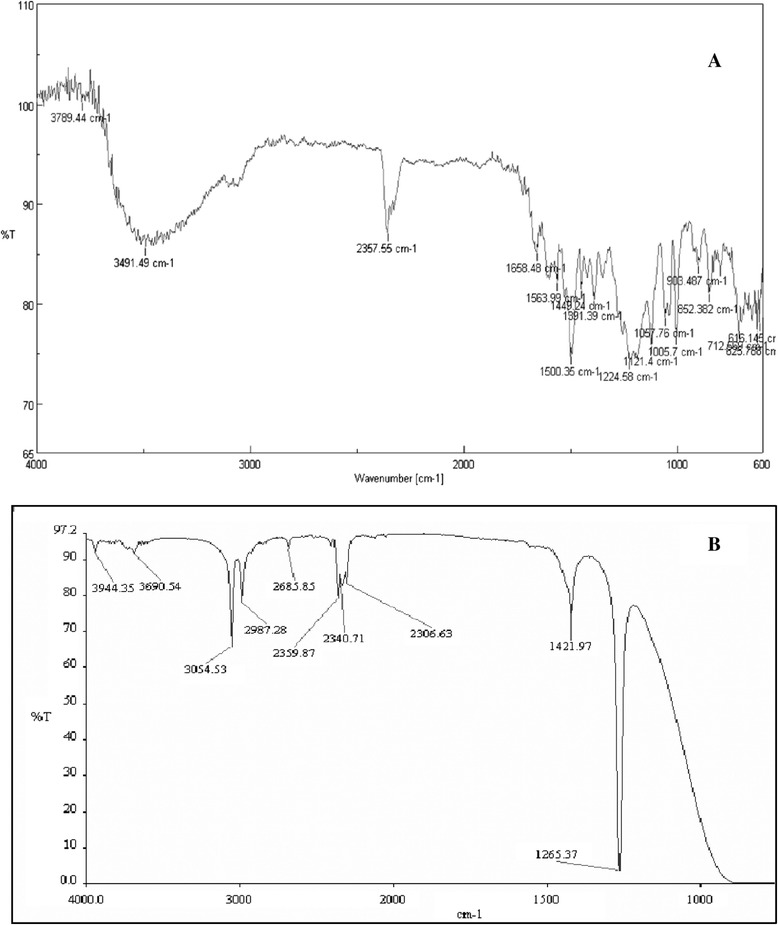
**FTIR analysis of products (extracted with diicholoromethane) formed by degradation of C.I. Direct Red 81: (A) at 0 h (control), (B) metabolites formed by** ***Enterococcus faecalis*** **YZ 66 after complete decolorization (1.5 h).**

The FTIR spectrum of a control dye and metabolites was compared. The spectrum of the control dye displayed a peak at 3789.44 cm^−1^ and 3491.49 cm^−1^ N-H stretching. The peak at 1658.48 cm^−1^ represents N = N symmetric stretch. A peak at 1563.99 cm^−1^ represents N-H bending. A peak at 1224.58 cm^−1^ represents C-O stretch band of phenol. The peaks at 1121.4 and 1057.76 cm^−1^ represents C-N stretch along with O = S = O symmetric stretch. The peaks at 616, 712 and 852.382 cm^−1^ represents C-H of substituted aromatics. FTIR spectrum of metabolites obtained after decolorization showed peaks at 3994.35 cm^−1^ and 3690.54 cm^−1^ represents phenolic –OH group, 3054.53 cm^−1^ represents = C-H stretch, 2987.28 cm^−1^ showed –C-H stretch and 1265.37 cm^−1^ represented –C-O stretching vibrations (Figure 5 A and B).

**Figure 6:**
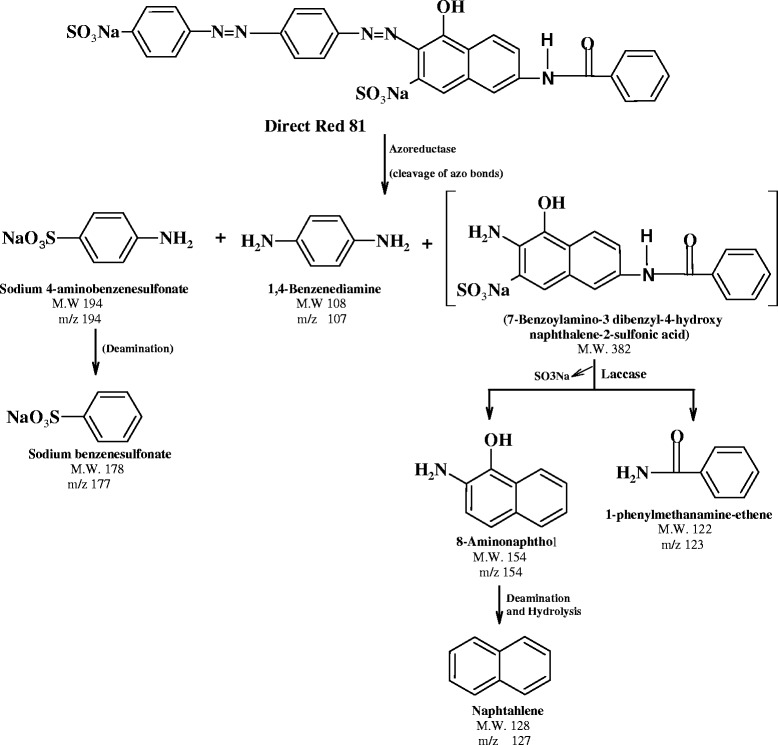
**Proposed pathway for biodegradation of C.I. Direct Red 81 by** ***Enterococcus faecalis*** **YZ 66.**

To verify the degradation products formed during dye decolorization by *E. faecalis*, GC–MS analysis was carried out. The low molecular weight aromatic compounds were produced from the degradation of Direct Red 81 by *E. faecalis*. Accordingly, the pathway for the degradation of Direct Red 81 is proposed as depicted in Figure 6, showing various steps involved in the degradation mechanism. However, very little is known about the nature of the degradation products formed in these reactions (Table 1) and the reaction mechanism about oxidoreductive enzymes. We propose that initially primary reductive cleavage in azo bond of Direct Red 81 results in the product such as, sodium-4-aminobenzenesulfonate, 1,4-benzenediamine and 7-benzylamino-3-dibenzyl-1-4-hydroxy naphthalene-2-sulfonic acid. Further deamination of sodium-4-aminobenzenesulfonate results into sodium benzenesulfonate with a mass peak of 178. Whereas the asymmetric cleavage of product 7-benzylamino-3-dibenzyl-1-4-hydroxy naphthalene-2-sulfonic acid by oxidative enzymes (laccase) resulted in the formation of 1-phenylmethanamine-ethene and 8-aminonaphthol as a products. Further deamination reaction resulting in the formation of low molecular weight compound such as naphthalene as a final product (Figure 6). Therefore, analytical studies confirmed the biodegradation of Direct Red 81 dye, in which the smaller molecular weight intermediates are formed by the consecutive action of oxidoreductive enzymes present in *E. faecalis*.

**Table 1:** **Mass spectrum data of degraded products of C.I. Direct Red 81 by Enterococcus faecalis YZ 66**

**Mass spectrum**	**Name of the product**
	Sodium-4-aminobenzenesulfonate (M.W.:194 m/z:192)
	1,4-benzenediamine (M.W.:108 m/z:107)
	Sodium benzenesulfonate (M.W.:178 m/z:177)
	8-aminonaphthol (M.W.:154 m/z:154)
	1-phenylmethanamine-ethene (M.W.:122 m/z:123)
	Naphthalene (M.W.:128 m/z:127)

### Phytotoxicity studies

Untreated or partially treated effluent may be disposed off in the water bodies and this water can be used for irrigation purpose. Thus it was found necessary to study phytotoxicity of the dye before and after degradation. The relative sensitivities towards the dye DR 81 and its degradation products in relation to *Sorghum vulgare* and *Phaseolus mungo* seeds were represented in the Table 2. There was no significant difference in the root and shoot length in case of the selected plants irrigated with the dye but in case of metabolites irrigated selected plants root and shoot length was significantly increased (*P ≤* 0.05) as compared to control. Phytotoxicity study showed good germination rate as well as significant growth in the plumule and radical for both the plants (*P* ≤ 0.05) in the metabolites extracted after decolorization as compared to dye sample. This indicates the detoxification of DR 81 by *E. faecalis*. Hence this indigenous bacterial strain could be a good biocatalyst for the treatment of textile dyes and effluent containing dyes.

**Table 2:** **Phytotoxicity studies of C.I. Direct Red 81 and its metabolites formed after biodegradation on** ***Phaseolus mungo*** **and** ***Sorghum vulgare***

	**For Direct Red 81**					
	***Phaseolus mungo***			***Sorghum vulgare***		
Parameters studied	Water	Direct red 81^a^	Extracted dye metabolites^a^	Water	Direct red 81^a^	Extracted dye metabolites^a^
Germination (%)	100	70	100	100	70	100
Shoot length (cm)	10.3	8.18	11.54	10.46	8.31	10.77
	±1.91	±1.70	±1.11	±1.12	±1.437	±1.31
Root length (cm)	5.11	4.55	7.23	6.64	5.042	8.0
	±1.35	±0.87	±1.13	±0.512	±0.692	±1.011

^a^400 ppm concentration.

Values are mean of three experiments, SEM (±), significantly different from the control (seeds germinated in distilled water) at *P < 0.05, **P < 0.001, by one-way analysis of variance (ANOVA) with Tukey–Kramer multiple comparisons test.

## Conclusions

This study demonstrates that isolated *Enterococcus faecalis* YZ 66 was able to degrade and detoxify the toxic sulfonated azo dye Direct Red 81 under static condition. Enzyme analysis indicated prime involvement of oxidoreductive enzymes in the decolorization process. The COD and BOD measurement showed mineralization of Direct Red 81 and phytotoxicity studies shows nontoxic residual metabolites. Analytical studies of extracted products confirmed the biodegradation of Direct Red 81 by *Enterococcus faecalis* YZ 66. A possible pathway for biodegradation of this dye was proposed with the help of GC-MS analysis. This strain also showed better colour removal of dye industry wastewater with significant reduction in COD and BOD and could be a potential strain for the treatment of textile dyestuffs and dye industry effluent by using appropriate bioreactor.

## Additional file

Additional file 1: Figure S1. UV-visible spectral scans of C.I. Direct Red 81 (50 mg/L) after complete decolorization by *Enterococcus faecalis* YZ 66. **Figure S2.** Decolorization and growth performance of C.I. Direct Red 81 by *Enterococcus faecalis* YZ 66. **Figure S3.** Effect of (A) pH and (B) temperature on decolorization performance of C.I. Direct Red 81 (50 mg/L) by using *Enterococcus faecalis* YZ 66. **Figure S4.** Effect of supplementation of different carbon and nitrogen sources on the decolorization of C.I. Direct Red 81 (50 mg/L) by using *Enterococcus faecalis* YZ 66. **Figure S5.** Effect of repeated addition of C.I. Direct Red 81 on decolorization performance by using *Enterococcus faecalis* YZ 66.
