# Supplementary material for: Decolorization and detoxification of sulfonated toxic diazo dye C.I. Direct Red 81 by Enterococcus faecalis YZ 66
Source: J Environ Health Sci Eng. 2014 Dec 24;12:151. doi: 10.1186/s40201-014-0151-1 (PMC4299565; doi:10.1186/s40201-014-0151-1)
Supplement: Additional file 1: Figure S1. — UV-visible spectral scans of C.I. Direct Red 81 (50 mg/L) after complete decolorization by Enterococcus faecalis YZ 66. Figure S2. Decolorization and growth performance of C.I. Direct Red 81 by Enterococcus faecalis YZ 66. Figure S3. Effect of (A) pH and (B) temperature on decolorization performance of C.I. Direct Red 81 (50 mg/L) by using Enterococcus faecalis YZ 66. Figure S4. Effect of supplementation of different carbon and nitrogen sources on the decolorization of C.I. Direct Red 81 (50 mg/L) by using Enterococcus faecalis YZ 66. Figure S5. Effect of repeated addition of C.I. Direct Red 81 on decolorization performance by using Enterococcus faecalis YZ 66. [file 40201_2014_151_MOESM1_ESM.doc]

Additional file

**Fig S1.** UV-visible spectral scans of C.I. Direct Red 81 (50 mg/L) after complete decolorization by *Enterococcus faecalis* YZ 66

**Fig S2.** Decolorization and growth performance of C.I. Direct Red 81 by *Enterococcus faecalis* YZ 66

**Fig S3.** Effect of (A) pH and (B) temperature on decolorization performance of C.I. Direct Red 81 (50 mg/L) by using *Enterococcus faecalis* YZ 66.

**Fig S4.** Effect of supplementation of different carbon and nitrogen sources on the decolorization of C.I. Direct Red 81 (50 mg/L) by using *Enterococcus faecalis* YZ 66

**Fig S5.** Effect of repeated addition of C.I. Direct Red 81 on decolorization performance by using *Enterococcus faecalis* YZ 66.

**Fig. S1**

**Fig. S2**

**(A)**

**(B)**

**Fig. S3**

**Fig. S4**

**Fig. S5**
